# A cleavage clock regulates features of lineage-specific differentiation in the development of a basal branching metazoan, the ctenophore *Mnemiopsis leidyi*

**DOI:** 10.1186/2041-9139-5-4

**Published:** 2014-01-31

**Authors:** Antje HL Fischer, Kevin Pang, Jonathan Q Henry, Mark Q Martindale

**Affiliations:** 1 Developmental Biology Unit, European Molecular Biology Laboratory Heidelberg, Meyerhof Strasse 1, Heidelberg 69117, Germany; 2 current address: Molecular and Cell Biology Department, Harvard University, 16 Divinity Avenue, Cambridge, MA 02138, USA; 3 Kewalo Marine Laboratory, Pacific Biosciences Research Center, University of Hawaii at Manoa, Honolulu, HI, USA; 4 current address: Sars International Centre for Marine Molecular Biology, Thormøhlensgt. 55, Bergen N-5008, Norway; 5 Department of Cell and Structural Biology, University of Illinois, 601 S. Goodwin Ave, Urbana, IL 61801, USA; 6 Whitney Lab for Marine Bioscience, Univ. Florida, 9505 Oceanshore Blvd, St, Augustine, FL 32080, USA

**Keywords:** Ctenophore, Comb jelly, Photocyte, Comb cell, Cytochalasin B, Puromycin, Actinomycin, Cell cycle arrest, Cell lineage

## Abstract

**Background:**

An important question in experimental embryology is to understand how the developmental potential responsible for the generation of distinct cell types is spatially segregated over developmental time. Classical embryological work showed that ctenophores, a group of gelatinous marine invertebrates that arose early in animal evolution, display a highly stereotyped pattern of early development and a precocious specification of blastomere fates. Here we investigate the role of autonomous cell specification and the developmental timing of two distinct ctenophore cell types (motile compound comb-plate-like cilia and light-emitting photocytes) in embryos of the lobate ctenophore, *Mnemiopsis leidyi*.

**Results:**

In *Mnemiopsis*, 9 h after fertilization, comb plate cilia differentiate into derivatives of the E lineage, while the bioluminescent capability begins in derivatives of the M lineage. Arresting cleavage with cytochalasin B at the 1-, 2- or 4-cell stage does not result in blastomere death; however, no visible differentiation of the comb-plate-like cilia or bioluminescence was observed. Cleavage arrest at the 8- or 16-cell stage, in contrast, results in the expression of both differentiation products. Fate-mapping experiments indicate that only the lineages of cells that normally express these markers in an autonomous fashion during normal development express these traits in cleavage-arrested 8- and 16-cell stage embryos. Lineages that form comb plates in a non-autonomous fashion (derivatives of the M lineage) do not. Timed actinomycin D and puromycin treatments show that transcription and translation are required for comb formation and suggest that the segregated material might be necessary for activation of the appropriate genes. Interestingly, even in the absence of cytokinesis, differentiation markers appear to be activated at the correct times. Treatments with a DNA synthesis inhibitor, aphidicolin, show that the number of nuclear divisions, and perhaps the DNA to cytoplasmic ratio, are critical for the appearance of lineage-specific differentiation.

**Conclusion:**

Our work corroborates previous studies demonstrating that the cleavage program is causally involved in the spatial segregation and/or activation of factors that give rise to distinct cell types in ctenophore development. These factors are segregated independently to the appropriate lineage at the 8- and the 16-cell stages and have features of a clock, such that comb-plate-like cilia and light-emitting photoproteins appear at roughly the same developmental time in cleavage-arrested embryos as they do in untreated embryos. Nuclear division, which possibly affects DNA-cytoplasmic ratios, appears to be important in the timing of differentiation markers. Evidence suggests that the 60-cell stage, just prior to gastrulation, is the time of zygotic gene activation. Such cleavage-clock-regulated phenomena appear to be widespread amongst the Metazoa and these cellular and molecular developmental mechanisms probably evolved early in metazoan evolution.

## Background

**Figure 1:**
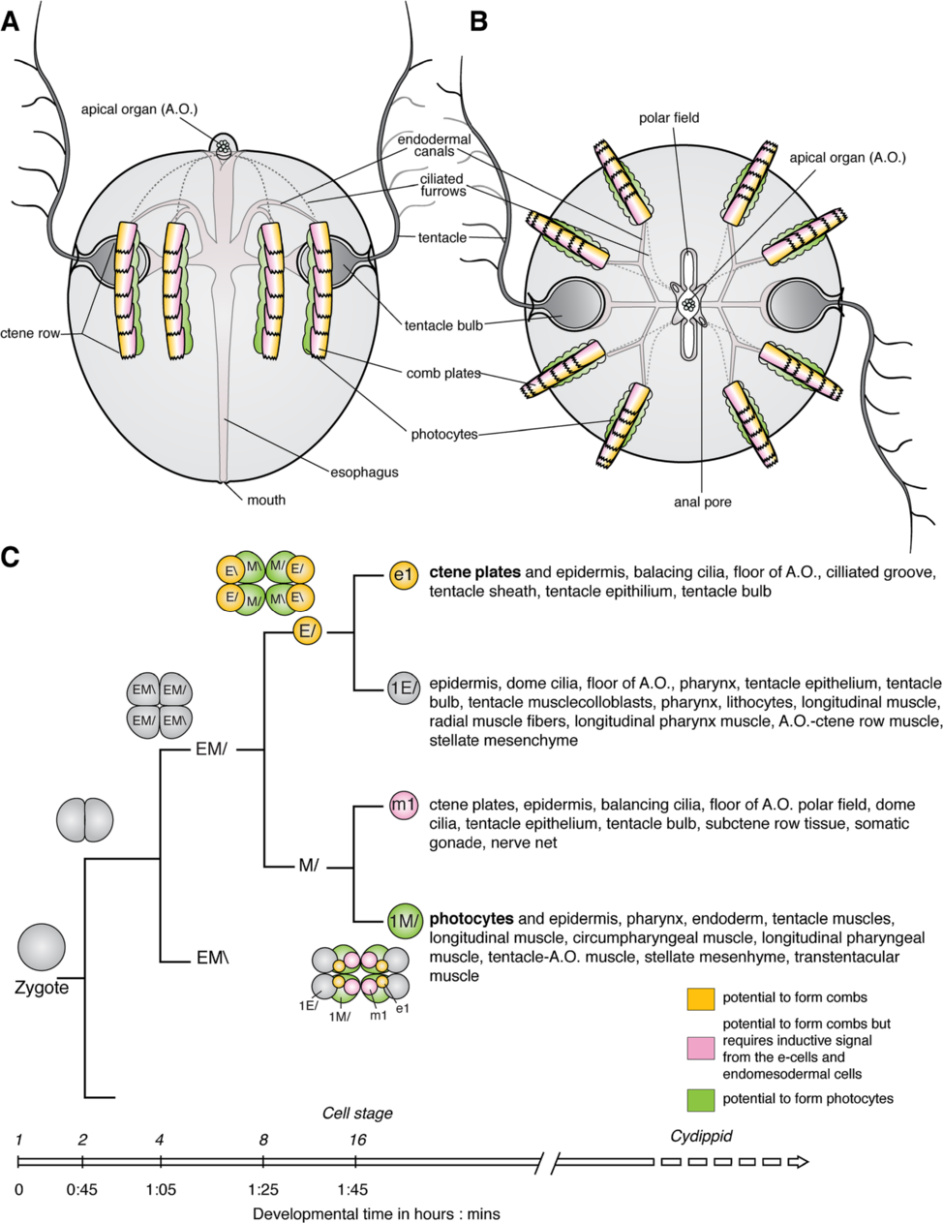
**Ctenophore development and cell lineage.** Ctenophore body plan, with **(A)** lateral view, aboral pole up and **(B)** aboral view. The comb plates are shown in yellow to indicate the contribution from e1 micromeres (yellow) and m1 micromeres (pink). The photocytes, are a derivative of the 2 M cells and associated with the endodermal canals underlying the comb rows [4,16]. **(C)** Simplified cell lineage fate map showing the e_1_, m_1_, 1E/ and 1 M/ micromere sublineages. For each of the different early cleavage stages there are diagrams showing the embryo from the aboral pole. Modified after [1,4,14].

Ctenophores are a monophyletic group of seemingly simple marine animals with distinct features, such as unique comb rows, body symmetry and stereotypic cleavage program [1-11]. The name Ctenophore means ‘comb-bearing’ and refers to eight rows of comb or ctene plates, each possessing thousands of motile cilia arranged in linear arrays to form small paddles [12]. Each comb plate in a ctene row beats in a highly synchronized manner and they are used to move the animal through the water column [12]. The oral-aboral axis is the major body axis of ctenophores and it is characterized by the mouth at one pole and the statocyst-containing apical organ at the opposite (aboral) pole [3,4,12] (Figure 1A, B). The view from the aboral pole shows that ctenophores comprise four highly similar quadrants, which are separated by two orthogonal planes – the tentacular plane and the esophageal or sagittal plane (Figure 1A) [4,13-15]. These planes are defined by axes of rotational symmetry around the oral-aboral axis because diagonally opposed quadrants are more identical to one another than adjacent quadrants [3-6][10,13,15].

Many ctenophores, including the lobate ctenophore *Mnemiopsis leidyi*, generate distinct light-producing photocytes during development, which migrate to locations subjacent to each of the eight comb rows. Genome sequencing of *Mnemiopsis* has revealed two genomic clusters containing ten distinct copies of photoproteins closely resembling the luciferase-type photoproteins found at the base of the Metazoa. *In situ* hybridization studies have shown that at least subsets of these mRNAs are expressed in photocytes prior to when these embryos are bioluminescent [16]. Thus, the presence or absence of bioluminescence is a strong indicator of the developmental fate of a differentiated photocyte.

**Figure 2:**
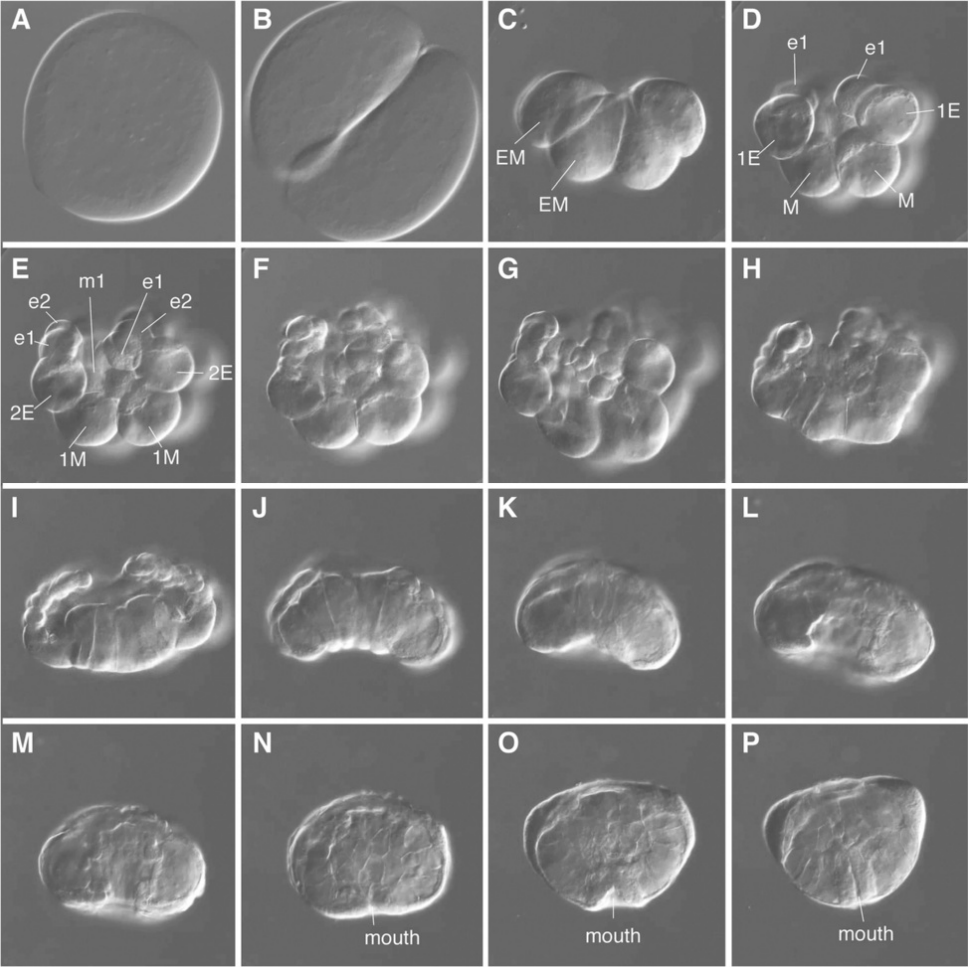
**Embryonic development of*Mnemiopsis leidyi.*** DIC images of developing *Mnemiopsis* embryos at various stages, beginning with a zygote in **(A)** until 9 hpf in **(P)**. **(A)** Zygote. **(B)** 2-cell stage. **(C)** 4-cell stage. **(D)** 8-cell stage. **(E)** 16-cell stage. **(F, G, H)** 32- to 60-cell stages. **(I, J, K, L)** Gastrulation. **(M, N, O, P)** Post-gastrulation. The aboral side is up. hpf, hours post fertilization. Additional file 1 shows the embryonic development of the same specimen in a time-lapse movie.

Ctenophore development is highly stereotypic and unique within the animal kingdom [1,4,5]. In the lobate ctenophore *Mnemiopsis leidyi*, embryos are generally fertilized at the time of spawning and cleavages are unipolar and holoblastic (Figure 1, and 2 Additional file 1) [4-6,10,17]. Subsequent rounds of division occur every 15 to 20 min at room temperature and the juvenile cydippid stage hatches from the egg membrane within 24 h after the first cleavage. The second cleavage, which is perpendicular to the first cleavage, gives rise to four similar-sized EM blastomeres [1,4,10,18]. The third cleavage is oblique and results in the formation of four E cells (end cells) and four M cells (middle cells) [1,4,10,14,18] (Figure 2 and Additional file 1). Each M blastomere undergoes two rounds of asymmetric division, giving rise to two small m micromeres (m_1_ and m_2_) towards the aboral pole and one M macromere towards the oral pole [1,4,14]. E blastomeres undergo three asymmetric cell divisions each, producing three small e micromeres (e_1_, e_2_ and e_3_) at the aboral pole and an E macromere at the oral pole [1,4,14]. The micromeres proliferate further and begin to envelop the macromeres during gastrulation via epiboly at approximately 3 to 4 hours post fertilization (hpf) [4,6] (Figure 2 and Additional file 1). Later during development, the macromeres, which are located at the oral pole, generate another set of oral micromeres [4]. Gastrulation is complete around 5 to 6 hpf. Ciliated comb cells appear by 9 hpf at the same time bioluminescence is detected. Cydippid stage juveniles hatch after 18 to 24 hpf [1,4,6,18].

The stereotyped cleavage program in ctenophores allows each blastomere to be identified and its fate followed by the injection of intracellular lineage tracers [1,4,7] (summarized in Figure 1C). For example, the mesoderm, including muscle, mesenchymal cells and photocytes, is generated by the micromeres born from endodermal precursors at the future oral pole [4] (summarized in Figure 1C). Early labeling experiments identified that the e_1_ micromeres give rise to the comb plate cilia [18,19]; however, later fate-mapping experiments were able to detect the m_1_ micromere’s contributions to the formation of comb plates (summarized in Figure 1C). Interestingly, when e_1_ micromeres are deleted, no comb plate cilia form, indicating that e_1_ micromeres are autonomously specified to give rise to comb plates while m_1_ micromeres require inductive signals [18,20].

Here, we investigate the role of the cleavage program in the segregation and expression of the developmental potential of two distinct cell types (motile comb-plate-like cilia and light-emitting photocytes) during the development of the ctenophore *Mnemiopsis*. Using an inhibitor of filamentous actin polymerization (cytochalasin B) to block cytokinesis (but not nuclear division), we show that the formation of differentiation markers associated with each of these cell types appears only after they have segregated into their own distinct lineages and they are never co-expressed in the same cells. Furthermore, using inhibitors of protein synthesis, transcription and DNA synthesis, we provide evidence for the existence of a cleavage clock that is based on the number of rounds of DNA synthesis (and possibly controlled by the nuclear-to-cytoplasmic ratio), which determines the temporal appearance of differentiation products in cleavage-arrested embryos.

## Methods

### *Mnemiopsis* collection and embryos

*Mnemiopsis leidyi* adults were collected from the National Oceanic and Atmospheric Administration jetty and Eel Pond in Woods Hole, MA, during June and July and from 13660 Deering Bay Dr, Coral Gables, FL 33158, during December. Adults were brought into the lab and induced to spawn as described previously [21]. Eggs were washed multiple times with 0.2 μm filtered seawater to remove any jelly or debris. Only batches in which a high percentage of embryos developed normally were used in the study. Fertilization in these animals occurs at spawning, so for developmental timing purposes this was designated as 0 hpf.

### Pharmaceutical inhibitors

After the embryos were collected, they were transferred by pipet to 24-well culture dishes for drug treatments. The following drugs were used: cytochalasin B (Sigma, St. Louis, MO, USA, C6762), actinomycin D (Sigma, St. Louis, MO, USA, A1410), puromycin (Sigma, St. Louis, MO, USA, P7255) and aphidicolin (Sigma, St. Louis, MO, USA, A0781). Stock solutions of cytochalasin (1 mg/ml), actinomycin (1 mg/ml) and aphidicolin (1 mg/ml) were made up in dimethyl sulfoxide, with aliquots stored at -20°C. A puromycin stock solution (12.5 mg/ml) was made up in distilled water and stored at -20°C. Freshly thawed aliquots were used in each experiment. Working solutions were made by diluting stock solutions with filtered seawater. Approximately 1 ml of solution was added to each well containing embryos.

### Hoechst staining and immunohistochemistry

The embryos were fixed for antibody staining in 4% paraformaldehyde and 0.02% glutaraldehyde, as previously described by Pang and Martindale [21]. Following fixation, the embryos were removed from their membranes by gentle pipetting, washed with PBS plus 0.2% Triton (PBT), blocked in 5% goat serum for 1 h and then incubated in anti-tyrosine tubulin (Sigma, T9028) overnight at 4°C. The embryos were washed six times for 30 min in PBT and incubated with the secondary antibody, goat anti-mouse conjugated to Alexa-594 (Invitrogen, Molecular Probes, Carlsbad, CA, USA), Alexa-488 phalloidin (Invitrogen, Molecular Probes, Carlsbad, CA, USA) and Hoechst 33342 (Invitrogen, Molecular Probes, Carlsbad, CA, USA) overnight at 4°C. Afterwards the embryos were washed twice for 5 min in PBS and mounted for imaging.

### Lineage tracing

After the embryos were collected, the vitelline membranes were mechanically removed using sharpened forceps. The de-membranated embryos were allowed to develop to the correct cleavage stage prior to injection with DiI, (Catalog No. D-282; Molecular Probes, Inc., Eugene, OR) a lipophilic membrane stain that diffuses laterally to stain the entire cell, as described in [4]. Following DiI injection, the embryos were carefully transferred into individual wells of a Terasaki plate (Nunc, Roskilde, Denmark) so that each embryo remained separate. Each well of the plate contained 10 μl of filtered seawater (controls) or cytochalasin B solution (1 μg/ml). The Terasaki plate was then stored in a humidified chamber to minimize evaporation.

### Imaging

The embryos were scored for the presence or absence of comb cells using a Stereo Discovery (Zeiss, Inc) or Axio Scope (Zeiss, Inc) under transmitted light. Live comb plate cells were imaged using time-lapse microscopy with time intervals of five to ten images per second. Normal *Mnemiopsis* development was recorded at three images per minute using DIC (Differential interference contrast). Visualization of bioluminescence was performed on an Axio Scope, using the GFP filter (38 HE Green Fluorescent Prot. filter, excitation BP 470/40 nm, emission BP 525/50 nm) to stimulate photoprotein emission and with 0.5 to 1 sec exposure times with an AxioCam Mr to capture faint signals. Prior to visualization, an embryo was kept undisturbed in darkness for 10 to 20 min to prevent activation and depletion of the photoprotein.

Confocal imaging after immunohistochemical staining was performed using a Zeiss 710 confocal microscope. Images were processed using Zen software (Zeiss, Inc) and Volocity (Improvision, Inc) to create 3D image reconstructions of confocal sections.

## Results

Cytochalasin B inhibits the polymerization of actin and thus prevents cytokinesis. During embryonic development, individual cleavages and specific developmental stages can be arrested by cytochalasin B, and thus the developmental potential of cells that were present at the time of arrest can be analyzed. We used cytochalasin B on zygotes, the 2-, 4-, 8- and 16-cell stages and at later time points up to the 60-cell stage (at 3, 3.5, 4, 5, 6, 7, 8 and 9 hpf). An overview of *Mnemiopsis* development is shown in Figure 2 and Additional file 1.

**Figure 3:**
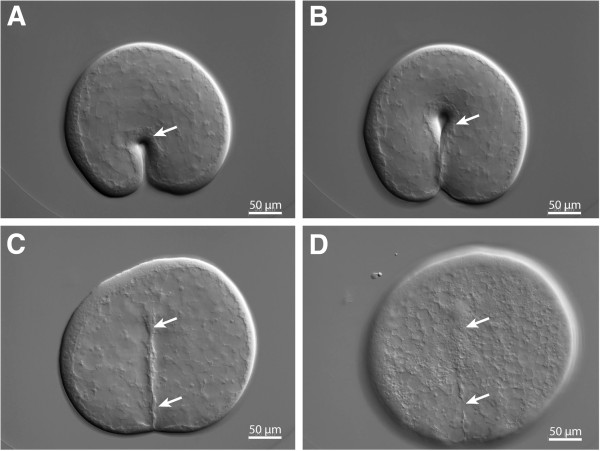
**Regression of the cleavage furrow (marked with arrows) after cytochalasin B treatment.** Cytochalasin was added during the first cleavage of a *Mnemiopsis* zygote. DIC images showing the ingressing cleavage furrow (arrow) **(A, B)**. Once cytochalasin B has been added, cleavage stops **(C)** and finally the cleavage furrow regresses **(D)**. Additional file 2 shows the regression of the cleavage furrow in a time-lapse movie in the same specimen.

**Figure 4:**
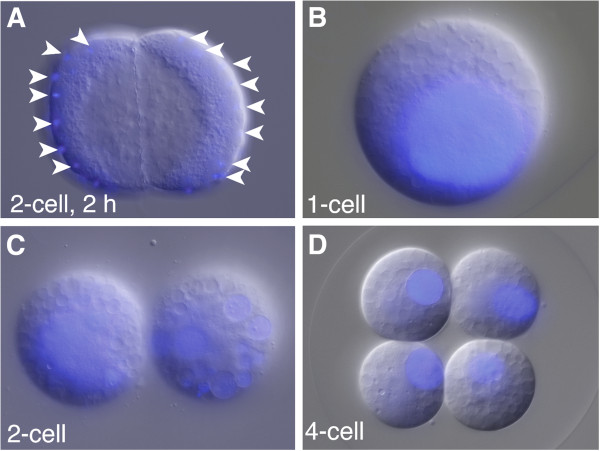
**Nuclear divisions in cleavage-arrested embryos continue.** Nuclei continue to divide after cytochalasin B treatment resulting in multiple nuclei in each cell, which later fuse into one or several large nuclei per cell. DNA staining with Hoechst (blue) merged with DIC images. **(A)** Embryo inhibited with cytochalasin B at the 2-cell stage. Multiple nuclei are visible along the periphery of the cells after 2 h of treatment. **(B)** A zygote arrested with cytochalasin B with one overly large nucleus. **(C)** Embryo inhibited with cytochalasin B at the 2-cell stage, with one large nucleus in the left cell and several large nuclei in the right cell. **(D)** Embryo inhibited with cytochalasin B at the 4-cell stage. Each cell has one large nucleus. Since all the embryos were inhibited prior to the 8-cell stage, none of them have differentiated comb cells.

Cytochalasin B treatment of *Mnemiopsis* embryos led to the immediate arrest of cytokinesis. If the embryo was undergoing cell division at the time of treatment, the cleavage furrow immediately began to regress and the blastomeres rounded up (Figure 3, Additional file 2). Throughout the cytochalasin B treatment, the blastomeres did not die and cell nuclei continued to divide as karyokinesis does not require actin polymerization [22]. For the first 4 to 5 h of treatment, the nuclei divided at the normal rate and remained in the periphery of the cell (Figure 4A), but after approximately 5 hpf, individual nuclei fused together into one or several large nuclei (Figure 4B, C, D). Cleavage-arrested blastomeres adhered tightly to each other until 7 to 8 hpf, after which individual cells tended to lose contact with one another. Although the normal configuration of the blastomeres was lost, they were still contained within the vitelline membrane, allowing for the identification of all descendants from an individually arrested embryo.

**Figure 5:**
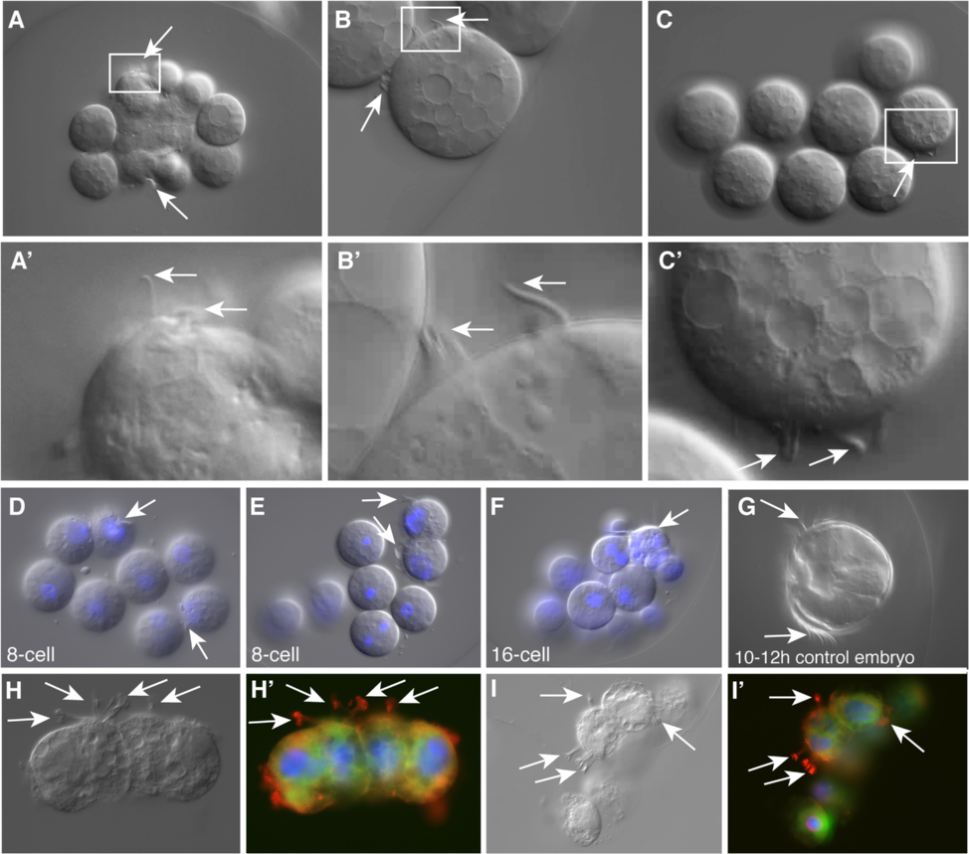
**Comb cells in cleavage-arrested embryos.** The arrows point to the cilia of the differentiated comb cells. **(A, B, C)** DIC images of cleavage-arrested embryos. **(A', B', C')** Close-up views of the rectangles in **(A, B, C)**. **(D, E, F)** DNA staining with Hoechst (blue) merged with DIC images of embryos inhibited with cytochalasin B at the 8-cell stage **(D, E)** or 16-cell stage **(F)**. **(G)** Control embryo at 10 to 12 hpf. DIC images **(H, I)** and z-projections of confocal images **(H', I')** of embryos stained with anti-tyrosinated tubulin (red) showing the cilia, Alexa-488 phalloidin (green) showing cell borders and Hoechst 33342 (blue) showing the nuclei. hpf, hours post fertilization. Additional file 3, Additional file 4, Additional file 5 are movies showing the motility of the cilia in the specimens shown in **A**, **B** and **C** respectively. Additional file 6 shows the movement of the cilia in the comb rows of the specimen shown in **G**.

While cytochalasin-treated zygotes and 2- and 4-cell stages did not show any visible sign of cell differentiation even after 24 hpf (Figure 4), surprisingly, 70% (31/44) of arrested embryos treated at the 16-cell stage (Figure 5F) and 83% (19/23) of embryos treated at the 32-cell stage had ctene-like cilia around 9 hpf. When treated at the 60-cell stage and onwards, nearly 100% of all arrested embryos formed ctene-like cilia. And when treated with cytochalasin B at the 8-cell stage, 26% (9/35) of the arrested embryos had two or more cells that formed motile comb-plate-like compound cilia (Figure 5, Additional file 3, Additional file 4, Additional file 5). These cilia were approximately the same size and formed at the same time as for untreated control embryos (Figure 5G, Additional file 6). A closer look at the ctene cells in treated embryos revealed that individual comb-plate-like cilia appeared morphologically normal with rows of cilia beating back and forth (Figure 5A, B, C, H, H', I, I', Additional file 3, Additional file 4, Additional file 5). Ctenophores make other kinds of ciliated structures (for example, dome cilia, sensory pegs, ciliated grooves and balancing cilia) but most of these are not motile, and none of them comprise compound cilia such as those made by the cleavage-arrested cells. Many individual cleavage-arrested cells formed multiple motile comb-plate-like cilia; these combs were not arranged in discrete rows as is the case during normal development [23] and their beating was not coordinated and appeared random with respect to one another and to neighboring cells. The cilia of the comb-plate-like generating cells were seen beating for up to 18 hpf and these cells quickly separated from the other cells in the embryos.

### Ctene cells in cleavage-arrested embryos are derived from e_1_ micromeres

**Figure 6:**
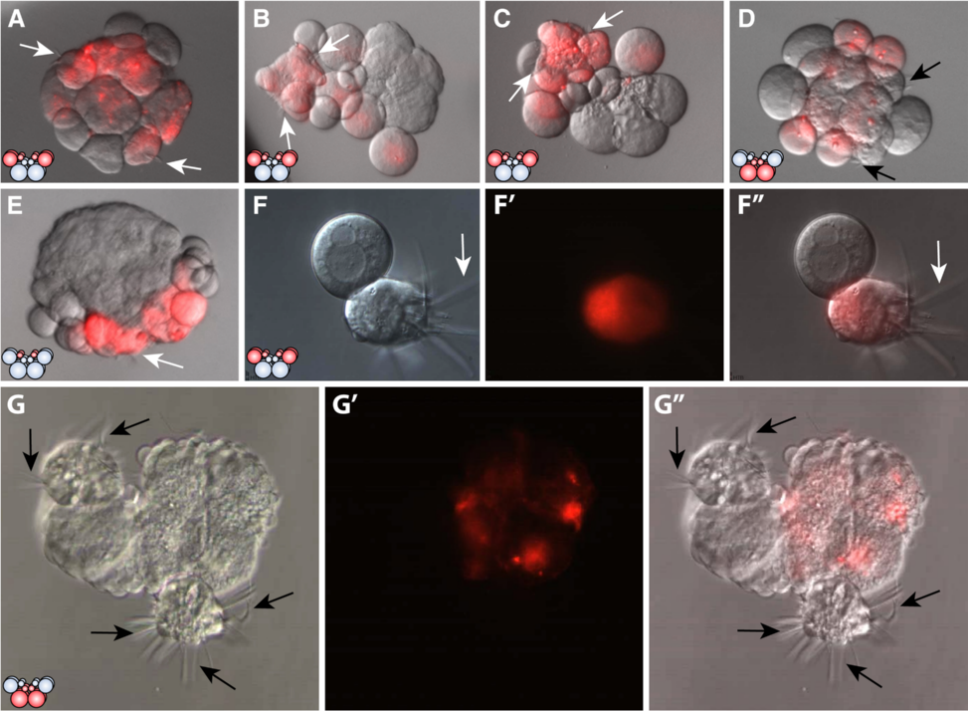
**Lineage tracing experiments in cleavage-arrested embryos show that e1 micromeres give rise to the comb-cells.** DIC images of cleavage-arrested embryos with DiI-labeling shown in red. White arrows point to labeled comb plates and black arrows point to unlabeled comb plates. The diagrams in the lower left corners show the stage when the embryo was inhibited with cytochalasin B and the appropriate DiI-labeled cells. **(A, B, C)** All four E macromeres were labeled at the 8-cell stage and the embryos were arrested at the 16-cell stage. Only labeled cells have combs. **(D)** All four M macromeres were labeled at the 8-cell stage and the embryos were arrested at the 16-cell stage. Only unlabeled cells form combs. **(E)** All four e_1_ micromeres were labeled and the embryos were arrested at the 16-cell stage. All comb cells are labeled. **(F, F'')** Part of an embryo that was arrested at the 16-cell stage. All E macromeres are labeled. **(F)** is a DIC image. **(F')** is the red channel and **(F'')** shows both images merged. The comb cell is clearly labeled. **(G, G', G'')** The embryo was arrested at the 16-cell stage. All four M macromeres are labeled. **(G)** is the DIC image and **(G')** the red channel. **(G'')** shows both images merged. All comb cells are unlabeled.

During normal *Mnemiopsis* development, ctene cells are formed by e_1_ micromeres and m_1_ micromeres [4]. However, while e_1_ micromeres can differentiate into comb-bearing cells autonomously, m_1_ micromeres require an inductive signal from the E lineage [1]. To determine which cell lineages were forming the comb cells in cleavage-arrested embryos, we utilized DiI labeling. Labeling of all four E blastomeres at the 8-cell stage and arresting the embryo at the 8- or 16-cell stage led to embryos in which all cells producing comb-plate-like cilia were labeled (Table 1, Figure 6A, B, C, F, F', F''). The same was true after labeling all four E macromeres at the 8-cell stage, allowing them to undergo one more cell division (to form DiI-labeled 1E macromeres and e_1_ micromeres) before arresting them at the 16-cell stage (Table 1, Figure 6F, F', F''). These experiments show that all cells that gave rise to comb-plate-like cilia in cleavage-arrested embryos were derived from the E lineage.

**Table 1:** **Overview of the cytochalasin treatment experiments on** ***Mnemiopsis leidyi*** **embryos**

**Developmental stage**	**Treatment**	**Observation**	**Conclusion**
8-cell stage	Cytokinesis arrested with cytochalasin B	50% have combs	All combs that were formed were labeled
16-cell stage		75% have combs	
32-cell stage		83% have combs	
8-cell stage	Cytokinesis arrested with cytochalasin B, labeling of all four E blastomeres	62% (8/13) have combs that are labeled	
		38% (5/13) did not form combs	
16-cell stage	Cytokinesis arrested with cytochalasin B at 16-cell stage, labeling of all four E blastomeres at the 8-cell stage	95/128 (74%) formed combs that are labeled	99% of embryos that formed combs were labeled, E cell lineage forms combs
		32/128 (25%) did not form combs	
		1/128 (0.8%) formed combs that are not labeled	
16-cell stage	Cytokinesis arrested with cytochalasin B at the 16-cell stage, labeling of all e_1_ micromeres at the 16-cell stage	27/36 (75%) formed combs that are labeled	100% of embryos that formed combs were labeled, e_1_ cells form combs
		9/36 (25%) did not form combs	
16-cell stage	Cytokinesis arrested with cytochalasin B at the 16-cell stage, labeling of all 1E macromeres at the 16-cell stage	No labeled combs	Only e_1_ cells at 16-cell stage form combs and E1 cells do not form combs
8-cell stage	Cytokinesis arrested with cytochalasin B at the 8-cell stage, labeling of all M blastomeres	19/27 (70%) formed combs that are not labeled	95% of the embryos formed combs that were not labeled and combs do not arise from M lineage cells
		7/27 (26%) did not form combs	
		1/27 (4%) formed combs that are labeled	
8-cell stage and 16-cell stage	Cytokinesis arrested with cytochalasin B at the 8-cell stage or 16-cell stage, all E blastomeres were labeled	Unlabeled cells have photoprotein	M cells can make photoprotein even when they are cell-cycle arrested

To rule out the possibility that DiI labeling could induce the cell fate of the comb-plate-like cilia, we labeled all four M blastomeres at the 8-cell stage or all four 1M macromeres and m_1_ micromeres at the 16-cell stage and arrested the embryos with cytochalasin B. In only one case (which was likely to have been generated from a misinjected cell), were the cells producing the comb-plate-like cilia labeled (Figure 6D, G).

To determine whether e_1_ cells specifically gave rise to the comb plates, we labeled all four e_1_ micromeres at the 16-cell stage and immediately afterwards treated the embryos with cytochalasin B. All of the resulting embryos formed cells producing comb-plate-like cilia that were labeled with DiI (Figure 6E, Table 1, Additional file 7). We also labeled all four 1E macromeres after the division of the E blastomeres into e_1_ and 1E at the 16-cell stage and cleavage arrested the embryos with cytochalasin B. None of the cells producing comb-plate-like cilia that formed were labeled (Table 1). These data demonstrate that only E lineage blastomeres at the 8-cell stage and e_1_ micromeres at the 16-cell stage give rise to comb-bearing cells. 1E macromeres, but not M lineage descendants, appear to be able to contribute to the formation of comb-plate-like cilia.

### Photocytes are specified by the M lineage in cleavage-arrested embryos

**Figure 7:**
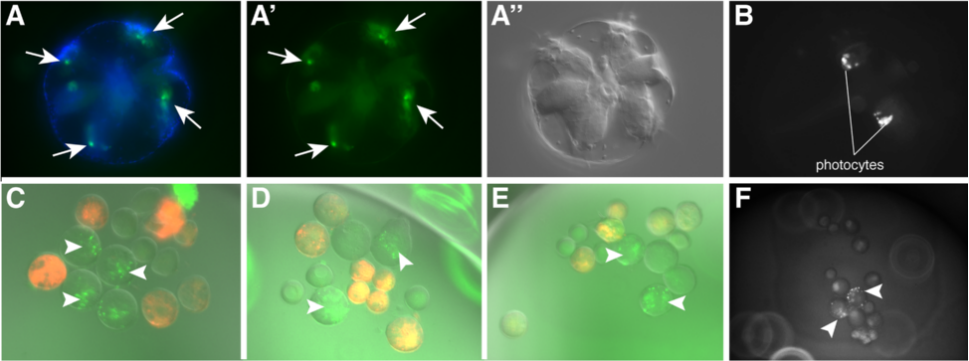
**Photocytes in control embryos and cleavage-arrested embryos. (A, A', A'')** In normal embryos, photocytes (white arrows) are visible at 9 to 10 hpf in the endoderm beneath the comb rows. In this live animal, viewed from the aboral pole, **(A)** shows nuclei in blue (Hoechst 33342 staining) overlaid with photoprotein fluorescence (green). **(A')** Photoprotein fluorescence as viewed under the GFP channel. **(A'')** DIC image of the same embryo shown in **(A)** and **(A')**. **(B)** Photoprotein fluorescence of a control animal. **(C, D, E)** E macromeres were labeled with DiI at the 8-cell stage and embryos were cleavage arrested at the 16-cell stage. Photoprotein is visible as green dots (arrowheads), in cells that are not labeled. **(F)** Photoprotein in an embryo that was cleavage arrested at the 16-cell stage. hpf, hours post fertilization. Additional files 8 and 9 show videos of the light-emitting photocytes of the specimens shown in figures **B** and **F**.

To determine whether the M lineage also gives rise to products specific to differentiated cells in cleavage-arrested *Mnemiopsis* embryos, we looked for the presence of bioluminescence or light production. In *Mnemiopsis*, bioluminescence is generated by the expression of luciferase-like photoproteins in a specific cell type, the photocytes [11,16]. Fate-mapping experiments have shown that during development, the 2M macromeres give rise to photocytes [4,16,24,25]. Photocytes are normally located in the endodermal canals underneath the comb rows [11,26] (Figure 7A, A', A'', B). To visualize the photoprotein, embryos were acclimatized to the dark and imaged under a GFP filter set on a Zeiss compound microscope (Additional file 8). The blue fluorescent light excites the photoprotein in these cells causing them to emit light at around 485 nm to 496 nm [16,27]. In living cydippids, the luminescence appeared as green specks and faded away within 1 to 3 sec, which can be seen by the naked eye and was recorded by a CCD camera.

In embryos that were treated with cytochalasin B at the 8- or 16-cell stage, we observed green bioluminescence in the cleavage-arrested blastomeres. This began at the same time as in control embryos at around 9 hpf (Additional file 9). Because DiI labeling of M blastomeres masked the weak florescence of the photoproteins in injected embryos, we labeled the E blastomeres at the 8-cell stage with DiI. Observation of DiI-injected E lineage cleavage-arrested embryos at the 8- or 16-cell stage revealed that the photoprotein was located in the unlabeled M (8-cell) and 1M macromeres (precursors to the 2M lineage) at the 16-cell stage (Figure 7C, D, E, F).

### Transcription and translation are required for the formation of comb-plate-like cilia in cleavage-arrested embryos

The differentiation of comb-plate-like cilia in E lineage cells and photoproteins in M lineage descendants in cleavage-arrested embryos suggests that developmental potential is faithfully segregated to the correct spatial lineages by the early cleavage program. However, it is unclear what key factors may be segregated into the lineages. To gain insight into the molecular nature of the determinants for the formation of comb-plate-like cilia, we used inhibitors of mRNA and protein synthesis to see if these classes of molecules could be responsible for the expression of these structures.

We treated embryos at various stages, beginning with zygotes up to the time that comb-plate-like cilia are normally generated in intact embryos (9 hpf), with 10 μg/ml of actinomycin D, an inhibitor of transcription [28,29]. Embryos that were treated up to 6 hpf, did not form comb plates (Table 2), suggesting that the localized determinant is not solely an mRNA (possibly maternal in nature) that is required for comb-plate formation. These results suggest that additional transcription is necessary for the comb plates to form in descendants of the E lineage.

**Table 2:** **Overview of the aphidicolin, actinomycin D and puromycin treatment experiments on** ***Mnemiopsis leidyi*** **embryos**

**Developmental stage**	**Treatment**	**Observation**	**Conclusion**
2-cell stage	Arrest of DNA synthesis for 2 h with aphidicolin, 2 μg/ml	No combs formed (0/30)	DNA synthesis is essential for comb formation.
16-cell stage			
32-cell stage			
60-cell stage		27/30 (90%) formed combs, comb formation was delayed by 2 h	
4 hpf		29/30 (97%) formed combs, comb formation was delayed by 2 h	
5 hpf		29/30 (97%) formed combs, comb formation was delayed by 2 h	
60-cell stage and 4 hpf	Arrest of DNA synthesis and cytokinesis with aphidicolin, 2 µg/ml, plus cytochalasin B, 1 to 2 μg/ml	Immediately arrested, single nuclei observed, neither combs nor photoprotein were formed in any embryo (0/30)	DNA synthesis is essential for comb formation even in cleavage-arrested embryos.
1- to 2-cell stage	Blocking of transcription with actinomycin D, 10 μg/ml	0/30 gastrulated, development arrests at 32- to 60-cell stage	Zygotic transcription begins around the 32- to 60-cell stage and the specification of the combs requires more than only maternal RNAs.
4- to 8-cell stage		0/30 gastrulated, development arrests at 32- to 60-cell stage	
16- to 32-cell stage		10/30 (33%) gastrulate, 20/30 (67%) arrest at 32- to 60-cell stage	
60-cell stage		28/30 (93%) gastrulate but no pharynx is formed, 2/30 (7%) do not gastrulate	
5 hpf (post-gastrulation)		No pharynx is formed	
2-cell stage	Arrest of translation with puromycin, 125 μg/ml, 25 μg/ml	0/30 formed combs	Comb protein is translated prior to 6 hpf, comb formation requires protein that is translated in the embryo and not maternally loaded.
16-cell stage		0/30 formed combs	
60-cell stage		0/30 formed combs	
6 hpf (post-gastrulation)		32/30 (97%) formed combs	

It is interesting to note that embryos that were treated at the 1-, 2-, 4-, 8- and 16-cell stages continued to divide normally and arrested only prior to gastrulation at about the 60-cell stage. Embryos that were treated with actinomycin D at the 32- or 60-cell stage divided only a few more times and then became arrested (Table 2). This suggests that if actinomycin D is inhibiting the bulk of mRNA synthesis, then zygotic transcription appears to begin at about the 32- or 60-cell stage and there are sufficient stores of maternal mRNAs to allow the embryo to reach these stages of development.

To block protein synthesis, we treated embryos at various stages with puromycin (25 to 125 μg/ml) [30]. Comb formation was only observed in embryos that were treated after 6 hpf (at 25 μg/ml puromycin), suggesting that the key determinant is not solely that a protein is required for comb formation, but that proteins must be translated between 5 and 6 hpf (Table 2).

### The differentiation of combs is regulated by the number of nuclear divisions

Cells producing ctene-like cilia and photocytes are formed at the same time in control embryos and in embryos treated with cytochalasin B at the 8-cell stage or later, suggesting that the embryo has a molecular timing mechanism that operates in the absence of cytokinesis. Because nuclear divisions appear to proceed normally in embryos treated with cytochalasin B (Figure 4), the number of nuclear divisions may be an important component of the timing mechanism. To test whether the timing of the formation of the comb-plate-like cilia is dependent on the number of nuclear divisions, we treated embryos with the DNA synthesis inhibitor aphidicolin (2 μg/ml) [31] at various cleavage stages up through 5 hpf (Table 2). When treated during early cleavage, up to the 32-cell stage, we did not observe the formation of any comb-plate-like cilia. When treated at the 60-cell stage, 4 hpf, and 5 hpf, we did observe comb formation, although it was delayed by approximately 2 to 3 h (12 hpf as opposed to 9 hpf). Embryos that were treated with aphidicolin at 5 hpf for 2 h and then removed from the drug, formed comb-plate-like cilia with a delay corresponding approximately to the length of the drug treatment. These results are consistent with the fact that the aphidicolin treatment is reversible, that DNA synthesis is able to resume following the rescue (for example, the drug is not lethal) and that the correct total lineage-specific DNA content is important for the timing of the formation of comb-plate-like cilia. We did not see any comb formation in embryos that were treated with both cytochalasin B and aphidicolin, even after 6 hpf. The double treatment with cytochalasin B and aphidicolin is not reversible and embryos that were treated for 2, 3 or 4 hpf did not recover and did not form comb cells. These results showed that that the combined effects of blocking DNA synthesis and cytokinesis are more detrimental than either drug alone and indicate that a critical number of nuclear divisions is essential.

## Discussion

We used cytochalasin B to inhibit cytokinesis in early ctenophore embryos to examine how developmental potential is partitioned into two distinct cell lineages: those giving rise to comb plates and those forming the photocytes. When cytokinesis is arrested in early *Mnemiopsis leidyi* embryos, treated embryos do not undergo apoptosis, but rather nuclear divisions continue and give rise to multinucleated cells. Cytochalasin B was used to study the segregation of developmental potential in *Mnemiopsis* by Freeman [18]. Freeman [18] inhibited certain divisions including the first, second or third cleavage or the second and third cleavage by short treatments with cytochalasin B. When only the first cleavage was inhibited by a brief exposure, most embryos developed like the control embryos but were delayed by one cleavage cycle [18]. However, when the second, third or second and third cleavages were reversibly blocked, the inhibited cleavages were skipped and the embryos continued the cleavage program with a reduced number of cells [18].

Surprisingly, as we demonstrate here, if cytokinesis is arrested at the 8-cell stage or later and is permitted to remain arrested for the following hours of development, comb-plate-like cilia and the photoprotein-mediated bioluminescence characteristic of photocytes form at the correct time in development compared with control embryos. Lineage-tracing experiments reveal that comb-plate-like cells are derived from E cells at the 8-cell stage and from the e_1_ micromeres at the 16-cell state. These are the same cell lineages that autonomously generate comb-plate-like cilia when isolated from the rest of the embryo [1]. Previous intracellular fate-mapping experiments showed that m_1_ micromere descendants also have the capacity to make comb-plate-like cilia [4] but M lineage descendants were not found to give rise to comb-plate-like cilia in cleavage-arrested embryos. Likewise, the bioluminescence characteristic of M-lineage-derived photocytes was only seen in M-lineage-derived cells in cleavage-arrested embryos. Earlier experiments showed that the m_1_ micromeres require inductive signals first from the e_11_ or e_12_ micromeres and later from endomesodermal cells derived from E and M macromeres before they form combs [14,20]. Our results support the finding that m_1_ micromeres require additional inductive signals from endomesodermal cells for comb formation, since no combs were formed by m_1_ micromeres in cleavage-arrested embryos. This supports the finding that the presence of e_1_ micromeres is not sufficient to induce comb formation in m_1_ micromeres [14,20].

Interestingly, neither comb-plate-like cilia nor bioluminescence was detected in cleavage-arrested zygotes or 2- or 4-cell stage embryos, suggesting that the segregation of distinct E and M lineages is crucial for the expression of the lineage-specific differentiation products. These results suggest that factors that are required for comb-plate and photocyte determination are already present and localized at the 8- and 16-cell stages and may have mutually exclusive activity.

### The segregation of developmental determinants

One of the fundamental processes of development is the localization of factors that are required to establish cell polarity, break symmetry and drive the asymmetric specification of cell fates. Experimental embryologists have provided evidence for the segregation of factors that determine specific cell fates in a diverse variety of different organisms (examples are summarized in [32-34]). In some embryos, segregated factors are asymmetrically localized maternally to the cell cortex such as the developmental potential required for gastrulation in echinoderm embryos [35-39] and for the establishment of dorso-ventral polarity in many embryos that undergo spiral cleavage [40-43], or the maternal gradients in insect eggs [44-46]. Other cytoplasmic factors are segregated actively at the time of fertilization as in ascidians [47-49], amphibians [50,51] and soil nematodes [52,53].

In ctenophore embryos, all evidence suggests that the asymmetrical localization of developmental potential is actively segregated by the cleavage process itself. Each of the early cell divisions leads to a definite asymmetric cell fate. The site of first cleavage gives rise to the oral-aboral axis [10]. Interestingly, a similar correlation between the site of the first cleavage and the site of gastrulation, and thus the formation of the oral-aboral axis, has been observed in the hydrozoan *Clytia hemisphaerica* (previously called *Phialidium gregarium*) [54]. Freeman [54] showed that the site of the first cleavage can be altered from the side of the polar body formation experimentally in ctenophores as well as in hydrozoans [10,54]. In contrast, the eggs of several sea urchin species are already polarized along the animal-vegetal axis at the time of fertilization (summarized in [55]). Starfish eggs and even ascidian eggs show a similar polarization [56,57]. Based on currently available data, it could be assumed that an irrevocable polarization of the unfertilized egg was an evolutionary novelty of bilaterians whereas in basally branching taxa, such as ctenophores and cnidarians, the egg is polarized by the position of the female pronucleus, which determines the site of the first cleavage, after fertilization [17]. However, a broader taxon sampling is desired to further support this hypothesis.

In ctenophores, the second division gives rise to the anal axis [4,15,19]. The third division separates the E and M lineages [4,18], and the fourth division segregates fates between micromere lineages (for example, comb rows) and macromere lineages (for example, photocyte cells) [4,18]. Consequently, when cytokinesis is inhibited, the factors remain associated with the proper lineages that would have generated the descendants during normal development. Interestingly, photoprotein formation and comb-plate formation never appeared to occur in the same cell. It is possible that the developmental determinants, which are required for comb-plate and photoprotein differentiation, inhibit each other and only after these factors have been spatially segregated can they be activated.

Although we have little information on the cell biological nature of the segregation process in ctenophores, centrosomes have been shown to be a causal factor in the segregation of factors that specify cell fates in other systems. In *Caenorhabditis elegans*, the sperm-derived centrosome, a complex of several proteins that acts as the microtubule-organizing center, breaks the symmetry of the oocyte and sets off a series of events that relies on maternally deposited proteins and eventually leads to the establishment of the anterior-posterior, dorsal-ventral and left-right body axes [58,59]. A surprisingly large number of mRNAs are localized to specific cell lineages in the snail *Ilyanassa*[60]. These mRNAs are associated with the centrosomes, and as the cells divide the mRNAs are subsequently distributed in an asymmetric manner to specific daughter cells [61]. Experimental evidence shows that these factors play an active role in the unique development of these cells [60,61]. It would be interesting to investigate whether a centrosome-dependent mechanism is also involved in the segregation of developmental factors in *Mnemiopsis*, where distinct developmental fates are decided at each of the early divisions.

### What is the nature of segregated developmental determinants?

Ctenophore development is rapid, with cleavages occurring every 15 to 20 min. The asymmetrical localization of proteins or mRNAs into distinct lineages during each division could be a simple way to distribute components quickly so as to distinguish different cell fates. Freeman [18] showed that the developmental determinants that are required for comb formation begin to be localized to the aboral region already during the 2-cell stage. Although crude and not gene specific, our experiments that inhibit transcription and translation suggest that the distributed determinants are not simply represented by the full set of comb-plate protein or comb-plate mRNA. Instead subsequent transcription and translation are required for comb formation. The nature of the components that are asymmetrically localized during the early cleavage stages in *Mnemiopsis* remains unknown.

In other systems, RNAs, proteins, protein complexes or a mix of these are the developmental determinants that are distributed unevenly during development and which break the symmetries. A well-known example is the localization of the protein Dishevelled – a key player of the Wnt/ β-catenin pathway – to one side of an unfertilized egg. This is one of the first symmetry-breaking events in many bilaterians [17,62,63]. Other well-known examples of proteins with asymmetric localizations are Miranda, Prospero and Staufen during *Drosophila* neuroblast division [64] and the Par proteins during *C. elegans* development [65].

Besides the localization of proteins or protein complexes, RNAs are often asymmetrically stored and thus establish cell polarity and determine the different daughter cell fates. One of the most famous examples of RNA localization is the localization of the *Oskar* mRNA, which is one of the key components used to establish polarity in the *Drosophila* oocyte during *Drosophila* oogenesis [66-69]. Other examples of RNA localization are *ASH1* in budding yeast, *bicoid* in *Drosophila* embryos, *Vg1* in *Xenopus* oocytes and *CamKIIa* in distal dendrites in mammalian neurons (summarized in [70]).

Future experiments will be needed to reveal the nature of the developmental determinants that are segregated during early ctenophore development.

### Temporal regulation of comb-plate formation: counting cell divisions

The regulation of growth and differentiation are key to developmental processes. Moreover, the timing of developmental events is a particularly important aspect of development. We showed that the differentiation of comb cells in *Mnemiopsis* embryos, which are cleavage arrested at the 8-cell stage or later, subsequently occurs at the same time as in untreated control embryos. Furthermore, our results show that comb-plate formation is blocked if DNA replication is inhibited by aphidicolin in addition to the inhibition of cytokinesis by cytochalasin B. This indicates that the number of nuclear division cycles or the amount of DNA (the nuclear-to-cytoplasmic ratio) appear to be important factors for comb-cell differentiation and not simply the amount of time that has elapsed since the third cleavage. As previously shown, after the second and/or third cleavage is blocked, *Mnemiopsis* embryos skip the respective cleavages and continue in a timely manner with their subsequent cleavage program, suggesting that a timing system determines the orientation of each division [18]. Once this process is activated, the cleavage plane is determined by this timing mechanism and cleavages do not follow a set order with respect to each prior cleavage division [18]. Since the inhibition of the first cleavage only causes a delay of the program, which otherwise occurs normally, Freeman [18] concluded that the timing mechanism is initiated with the completion of the first cleavage.

Embryos that undergo exact numbers of cell divisions are known from a broad range of developmental systems; however, little is understood about the underlying mechanisms [71-75]. A stunning example is the highly stereotypic cell division pattern of the ventral germ band in malacostracan crustaceans. In *Cherax destructor*, for example, all ectoteloblasts undergo exactly 15 rounds of asymmetric cell division and give birth to ectodermal blast cells, which undergo two distinct divisions each [74].

Fundamental experiments by Whittaker showed that ascidian embryos developed muscle-specific acetylcholinesterase and brain pigment cell tyrosinase in specific blastomeres even in cleavage-arrested embryos and concluded that specific positional information is differentially segregated during early development [76]. Since then several studies have employed cytochalasin B to investigate the cell fate specification and cell lineage of ascidian embryos (for example, [77-80]). Satoh and Ikegami [75] performed a series of experiments combining cytochalasin B and aphidicolin in ascidian embryos, showing that future muscle cells must undergo eight cell-division cycles before they start expressing the muscle cell lineage marker acetylcholinesterase. Nevertheless, the question of how cells ‘count’ the number of cell cycles remains unanswered.

A number of counting mechanisms have been suggested for this phenomenon. Based on the observation that genomic DNA is highly methylated in zygotes and gradually demethylated during development, which tends to de-repress transcriptional activity in mammals [81], Kataoka *et al*. [73] suggested that changes in DNA methylation could be involved in keeping count of the number cell cycles in ascidians. However, their own results do not support this hypothesis as they were unable to detect any changes in DNA methylation [73]. Another possible explanation is that a specific ratio of DNA to cytoplasm is required. This ratio is crucial during early *Xenopus* development, where zygotic transcription is initiated after 11 to 12 rounds of cell division [82]. The required ratio of DNA-to-cytoplasmic volume can also be acquired when cytokinesis is blocked with cytochalasin B but DNA replication is allowed to continue further [82]. In contrast, in *C. elegans*, the timing of gut marker gene expression only depends on an early period of DNA synthesis until the 8-cell stage when the gut is clonally established [83]. Subsequent rounds of cell divisions, which usually occur after the 8-cell stage, can be inhibited without preventing the expression of the marker gene at a later stage, thus this is independent of the DNA-to-cytoplasmic ratio or the number of DNA synthesis rounds [83].

Future studies are required to unravel the mechanism that regulates the timing of cell differentiation in ctenophores, whether this is the ratio of DNA to cytoplasm, DNA methylation, protein degradation, telomeric alterations, a combination of different factors or an entirely different mechanism that is currently unknown.

### The onset of zygotic transcription

Early developmental events are regulated by gene products, which may be provided maternally [84]. The activation of the zygotic genome marks the maternal-to-zygotic transition (MZT), which was first described for *Xenopus*[82] and has since been studied in many model organisms [84]. It is estimated that around 40% (in mice) to 75% (in sea urchins) of all protein coding genes are represented as maternal mRNAs and are degraded throughout early development [85,86].

Other than bilaterians, the onset of zygotic transcription has only been described in cnidarians. In *Clytia hemisphaerica*, zygotic transcription starts at the late blastula to early gastrula stage [63,87]. The sea anemone *Nematostella vectensis* exhibits an upregulation of gene expression around 10 hpf [88], at a time in development when cell division is asynchronous [89].

We used actinomycin D to inhibit transcription and we showed that treatment prior to the 60-cell stage did not visibly affect development until the animal reached the 60-cell stage, suggesting that maternally loaded transcripts are sufficient to support the early cleavage program up to this stage but not subsequent development or differentiation. These results suggest that the onset of zygotic transcription starts at about the 60-cell stage, just prior to gastrulation. The rapid early development of ctenophores also suggests that there may be little time for the transcription of new messages until the cell cycle began to slow down. Future experiments using labeled nucleotides will help to determine the precise onset of zygotic transcription in *Mnemiopsis.*

The transition from maternal to zygotic transcription is most likely an ancient feature in animal evolution and might be tightly linked with the evolution of multicellularity and sexual reproduction. If ctenophores are the sister group of all metazoans, as suggested by recent studies [90,91], one could conclude that the inclusion of the MZT is part of the metazoan ground pattern. However, so far data about the MZT in sponges are not available and the phylogenetic position of ctenophores is still very much under debate [92]. There are currently several contradictory hypotheses, including: (a) ctenophores and cnidarians are sister groups [93-95] and (b) ctenophores are the sister group to all bilaterians [96]. Recent developmental studies support the idea that ctenophores lack several characters that are shared between cnidarians and bilaterians [13,97-101] and thus they support the idea that (c) ctenophores are the sister group to all remaining eumetazoans [102] or even, as mentioned above, that (d) ctenophores are the sister group to all metazoans [90,91].

A phylogenetic comparison of maternally loaded proteins and the molecular regulation of the maternal-to-zygotic transition in bilaterian and non-bilaterian taxa might provide further insight into the evolution of sexual reproduction and life history.

## Conclusions

**Figure 8:**
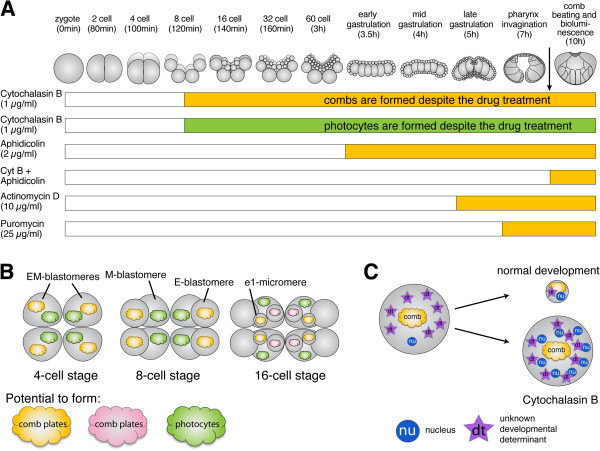
**Summary of mechanisms controlling the development of comb plates and photocytes. (A)** Overview of the normal embryonic development of *Mnemiopsis* and the different treatments with cytochalasin B, aphidicolin, cytochalasin B plus aphidicolin, actinomycin D and puromycin. The black arrow indicates when combs form during normal development. The yellow part of each bar indicates when a treatment starts that permits comb formation, and the green part indicates when a treatment starts that permits photocyte formation, in those cases where it has been tested. The white part of each bar indicates the developmental period, when treatment with the inhibitor blocks comb or photocyte formation. **(B)** Overview of the segregation of developmental determinants throughout early cleavages. All embryos are shown from the aboral pole. At the 4-cell stage, the developmental determinants are not yet segregated. At the 8-cell stage, the M blastomeres inherit factors that specify the photocyte lineage (green clouds labeled with ‘photo’) and the E blastomeres inherit factors that specify the comb-cell lineage (orange clouds labeled with ‘comb’). At the 16-cell stage, factors that specify the comb-cell lineage are segregated to the e_1_ micromeres. The m_1_ micromeres can form combs but they require an inductive signal from the e cells (pink clouds) in addition to signals provided by the endomesoderm [20]. **(C)** Schematic overview of changes DNA to cytoplasmic ratio during normal development and embryos treated with cytochalasin B, which is a potential mechanism for counting the number of cell divisions.

As in other animals with a mosaic development, embryos of the comb jelly *Mnemiopsis leidyi* show differentiation of selected cell fates even when cytokinesis is arrested using cytochalasin B during the course of early embryogenesis [48,77,78,103,104]. An overview is given in Figure 8. We demonstrated that embryos arrested prior to the 8-cell stage do not show visible signs of cell fate differentiation from either the E or M lineages. However, if cytokinesis is blocked at the 8-cell stage or later, comb-plate-like cilia, which are derived autonomously from the E lineage, and the bioluminescence characteristic of photocytes form at the correct time compared with control embryos. Treatment with the DNA synthesis inhibitor aphidicolin reveals that the number of nuclear divisions is essential for the proper timing of the differentiation of the comb-plate-like cilia. Lineage tracing experiments show that cells producing comb-plate-like cilia are formed autonomously from the E lineage, particularly the e_1_ micromeres, and not any of the M or m_1_ lineages. Likewise, bioluminescence is only observed in the M lineage and specifically in M macromeres. In addition, we showed that developmental determinants that are required to generate motile comb-plate-like cilia and photoprotein expression are already present and localized at the 8-cell stage and further localize to the appropriate cells in the subsequent cell divisions. Further studies are required to unravel the nature of the developmental determinants and which mechanisms are used to segregate them. The timed inhibition of transcription and translation with actinomycin D and puromycin, respectively, shows that both transcription and translation are required for comb cells to form, suggesting that not all the necessary factors are maternally deposited. While these inhibitor studies are not definitive gene-specific approaches, they do provide insight into the potential nature of materials that are differentially segregated into different embryonic lineages. Finally, we presented evidence that suggests that zygotic transcription in *Mnemiopsis* begins around the 60-cell stage, just before the onset of gastrulation.

## Abbreviations

hpf: hours post fertilization; MZT: Maternal-to-zygotic transition; PBS: Phosphate-buffered saline; PBT: PBS plus 0.2% Triton; DIC: Differential interference contrast; AO: Apical organ.

## Competing interests

The authors declare that they have no competing interests.

## Authors’ contributions

AHLF, KP, JQH and MQM conceived the study and analyzed the data. AHLF and KP conducted and analyzed the drug treatments and produced the figures. KP analyzed the immunohistochemistry, produced the confocal micrographs and recorded the time-lapse images. JQH and MQM performed the microinjection. KP, JQH and MQM imaged the injected embryos. AHLF wrote the first draft of the manuscript. All other authors helped to complete and edit the manuscript. All authors read and approved the final manuscript.

## Supplementary Material

Additional file 1 **Normal embryonic development of *Mnemiopsis leidyi*.** DIC microscopy time-lapse movie of developing Mnemiopsis embryos beginning with a zygote until 12 hpf. The aboral side is up. The animal shown is the same as in Figure 2A-P. Click here for file

Additional file 2 **Regression of the cleavage furrow in a *Mnemiopsis* embryo after cytochalasin B treatment.** Cytochalasin B was added during the first cleavage of a Mnemiopsis zygote. DIC microscopy time-lapse showing the ingression of the cleavage furrow. Once cytochalasin B has been added, cleavage stops and the cleavage furrow regresses. The animal shown is the same as in Figure 3A-D. Click here for file

Additional file 3 **Cleavage-arrested embryo with beating cilia at 2 comb cells.** DIC microscopy movie showing the beating cilia on comb-cells in a cleavage-arrested embryo. The embryo shown is the same one as in figure 5A and 5A'. Click here for file

Additional file 4 **Close-up on one a comb cell with beating cilia in a cleavage-arrested embryo.** DIC microscopy movie showing the beating cilia on comb-cells in a cleavage-arrested embryo. The embryo shown is the same one as in figure 5B and 5B'. Click here for file

Additional file 5 **Cleavage-arrested embryo with beating cilia at a comb cells.** DIC microscopy movie showing the beating cilia on comb-cells in a cleavage-arrested embryo. The embryo shown is the same one as in figure 5C and 5C'. Click here for file

Additional file 6 **Control embryo at around 10-12 hpf.** DIC microscopy movie showing the beating cilia of the comb cells, which are well visible on both sides of the embryo. The embryo shown is the same one as in figure 5G. Click here for file

Additional file 7 **Cleavage-arrested embryo with beating cilia and labeled e1-cell lineage.** DIC microscopy movie in combination with fluorescent light showing an embryo in which the e1-cells were DiI labeled at the 16-cell stage. The embryo was arrested with cytochalasin B at the 32-cell stage. Please note that only labeled cells bear cilia. The embryo shown is the same one as in Figure 6E. Click here for file

Additional file 8 **Bioluminescence in photocytes of a control embryos.** Light microscopy movie showing light emission from the photocytes that was stimulated by a brief illumination with fluorescent light. The photocytes are well visible on either side of the embryo. The animal shown is the same one as in Figure 7B. Click here for file

Additional file 9 **Bioluminescence in photocytes of a cleavage arrested embryos.** Light microscopy movie showing light emission from the photocytes that was stimulated by a brief illumination with fluorescent light. Those cells that contain the bioluminescent protein are photocytes. The embryo shown is the same one as in Figure 7C. Click here for file

## References

[B1] Martindale MQ Henry JQ Reassessing embryogenesis in the Ctenophora: the inductive role of e1 micromeres in organizing ctene row formation in the 'mosaic' embryo, *Mnemiopsis leidyi* Dev 1997 124 10 1999 2006 10.1242/dev.124.10.1999 9169846

[B2] Henry JQ Martindale MQ Evolution of cleavage programs in relationship to axial specification and body plan evolution Biol Bull 1998 195 3 363 366 10.2307/1543148 28297612

[B3] Martindale MQ Finnerty JR Henry JQ The Radiata and the evolutionary origins of the bilaterian body plan Mol Phylogenet Evol 2002 24 3 358 365 10.1016/S1055-7903(02)00208-7 12220977

[B4] Martindale MQ Henry JQ Intracellular fate mapping in a basal metazoan, the ctenophore *Mnemiopsis leidyi*, reveals the origins of mesoderm and the existence of indeterminate cell lineages Dev Biol 1999 214 243 257 10.1006/dbio.1999.9427 10525332

[B5] Martindale MQ Henry JQ The development of radial and biradial symmetry: the evolution of bilaterality Am Zool 1998 38 4 672 684

[B6] Martindale MQ The ontogeny and maintenance of adult symmetry properties in the ctenophore. *Mnemiopsis mccradyi* Dev Biol 1986 118 2 556 576 10.1016/0012-1606(86)90026-6 2878844

[B7] Martindale M Henry J Development and regeneration of comb plates in the ctenophore *Mnemiopsis leidyi* Biol Bull 1996 191 2 290 292 10.1086/BBLv191n2p290 29220260

[B8] Driesch H Morgan TH Zur Analysis der ersten Entwickelungsstadien des Ctenophoreneies Archiv für Entwicklungsmechanik der Organismen 1895 2 2 204 215 10.1007/BF02084247

[B9] Chun C Die Ctenophoren des Golfes von Neapel und der angrenzenden Meeres-Abschnitte: eine Monographie Fauna und Flora des Golfes von Neapel 1880 1 1 311

[B10] Freeman G The establishment of the oral-aboral axis in the ctenophore embryo J Embryol Exp Morphol 1977 42 1 237 260

[B11] Freeman G Reynolds GT The development of bioluminescence in the ctenophore *Mnemiopsis leidyi* Dev Biol 1973 31 1 61 100 10.1016/0012-1606(73)90321-7 4150750

[B12] Tamm SL Tamm S Ciliary reversal without rotation of axonemal structures in ctenophore comb plates J Cell Biol 1981 89 3 495 509 10.1083/jcb.89.3.495 6114102 PMC2111786

[B13] Pang K Ryan JF Mullikin JC Baxevanis AD Martindale MQ Genomic insights into Wnt signaling in an early diverging metazoan, the ctenophore *Mnemiopsis leidyi* EvoDevo 2010 1 1 10 10.1186/2041-9139-1-10 20920349 PMC2959043

[B14] Henry JQ Martindale MQ Inductive interactions and embryonic equivalence groups in a basal metazoan, the ctenophore *Mnemiopsis leidyi* Evol Dev 2004 6 17 24 10.1111/j.1525-142X.2004.04001.x 15108814

[B15] Martindale M Henry J Diagonal development: establishment of the anal axis in the ctenophore *Mnemiopsis leidyi* Biol Bull 1995 189 2 190 192 10.1086/BBLv189n2p190 27768492

[B16] Schnitzler CE Pang K Powers ML Reitzel AM Ryan JF Simmons D Tada T Park M Gupta J Brooks SY Blakesley RW Yokoyama S Haddock SHD Martindale MQ Baxevanis AD Genomic organization, evolution, and expression of photoprotein and opsin genes in *Mnemiopsis leidyi*: a new view of ctenophore photocytes BMC Biol 2012 10 1 107 10.1186/1741-7007-10-107 23259493 PMC3570280

[B17] Martindale MQ Hejnol A A developmental perspective: changes in the position of the blastopore during bilaterian evolution Dev Cell 2009 17 2 162 174 10.1016/j.devcel.2009.07.024 19686678

[B18] Freeman G The role of cleavage in the localization of developmental potential in the Ctenophore *Mnemiopsis leidyi* Dev Biol 1976 49 1 143 177 10.1016/0012-1606(76)90264-5 943342

[B19] Reverberi G Ortolani G On the origin of the ciliated plates and of the mesoderm in the ctenophores Acta Embryol Morph Exp 1963 6 175 190

[B20] Henry JQ Martindale MQ Multiple inductive signals are involved in the development of the ctenophore *Mnemiopsis leidyi* Dev Biol 2001 238 40 46 10.1006/dbio.2001.0401 11783992

[B21] Pang K Martindale MQ Comb jellies (Ctenophora): a model for basal metazoan evolution and development Cold Spring Harb Protoc 2008 2008 11 106 pdb. emo 10.1101/pdb.emo106 21356709

[B22] Sanger J Holtzer H Cytochalasin B: effects on cell morphology, cell adhesion, and mucopolysaccharide synthesis Proc Natl Acad Sci 1972 69 1 253 257 10.1073/pnas.69.1.253 4257818 PMC427586

[B23] Tamotsu S Samejima M Suzuki N Morita Y Three-dimensional reconstruction of serotonin-immunoreactive photoreceptors in the pineal organ of the river lamprey, *Lampetra japonica* Biol Signals 1997 6 4–6 184 190 9500655 10.1159/000109128

[B24] Haddock SH Case JF Not all ctenophores are bioluminescent: *Pleurobrachia* Biol Bull 1995 189 3 356 362 10.2307/1542153 29244577

[B25] Moore AR Luminescence in *Mnemiopsis* J Gen Physiol 1924 6 4 403 412 10.1085/jgp.6.4.403 19872081 PMC2140645

[B26] Anctil M Ultrastructure of the luminescent system of the ctenophore *Mnemiopsis leidyi* Cell Tissue Res 1985 242 2 333 340

[B27] Ward WW Seliger H Properties of mnemiopsin and berovin, calcium-activated photoproteins from the ctenophores *Mnemiopsis* species and *Beroe ovata* Biochemistry 1974 13 7 1500 1510 10.1021/bi00704a028 4819763

[B28] Perry RP Kelley DE Inhibition of RNA synthesis by actinomycin D: characteristic dose–response of different RNA species J Cell Physiol 1970 76 2 127 139 10.1002/jcp.1040760202 5500970

[B29] Bensaude O Inhibiting eukaryotic transcription. Which compound to choose? How to evaluate its activity? Transcription 2011 2 3 103 108 10.4161/trns.2.3.16172 21922053 PMC3173647

[B30] Nathans D Puromycin inhibition of protein synthesis: incorporation of puromycin into peptide chains Proc Natl Acad Sci U S A 1964 51 4 585 10.1073/pnas.51.4.585 14166766 PMC300121

[B31] Ikegami S Taguchi T Ohashi M Oguro M Nagano H Mano Y Aphidicolin prevents mitotic cell division by interfering with the activity of DNA polymerase-α Adv Physiol Educ 1978 275 458 460 10.1038/275458a0 692726

[B32] Johnstone O Lasko P Translational regulation and RNA localization in *Drosophila* oocytes and embryos Annu Rev Genet 2001 35 1 365 406 10.1146/annurev.genet.35.102401.090756 11700288

[B33] Davidson EH How embryos work: a comparative view of diverse modes of cell fate specification Development 1990 108 3 365 389 2187672 10.1242/dev.108.3.365

[B34] Strome S Lehmann R Germ versus soma decisions: lessons from flies and worms Science 2007 316 5823 392 393 10.1126/science.1140846 17446385

[B35] Angerer LM Angerer RC 4 patterning the sea urchin embryo: Gene regulatory networks, signaling pathways, and cellular interactions Curr Top Dev Biol 2003 53 159 198 12509127 10.1016/s0070-2153(03)53005-8

[B36] Weitzel HE Illies MR Byrum CA Xu R Wikramanayake AH Ettensohn CA Differential stability of β-catenin along the animal-vegetal axis of the sea urchin embryo mediated by *dishevelled* Development 2004 131 12 2947 2956 10.1242/dev.01152 15151983

[B37] Leonard JD Ettensohn CA Analysis of *dishevelled* localization and function in the early sea urchin embryo Dev Biol 2007 306 1 50 65 10.1016/j.ydbio.2007.02.041 17433285 PMC2697034

[B38] Kumburegama S Wijesena N Wikramanayake AH Detecting expression patterns of Wnt pathway components in *Nematostella vectensis* embryos Meth Mol Biol 2008 469 55 67 10.1007/978-1-60327-469-2_6 19109703

[B39] Logan CY Miller JR Ferkowicz MJ McClay DR Nuclear beta-catenin is required to specify vegetal cell fates in the sea urchin embryo Development 1999 126 2 345 357 9847248 10.1242/dev.126.2.345

[B40] Van Den Biggelaar JAM Development of dorsoventral polarity and mesentoblast determination in *Patella vulgata* J Morphol 1977 154 157 186 10.1002/jmor.1051540111 915947

[B41] Martindale MQ Doe CQ Morrill JB The role of animal-vegetal interaction with respect to the determination of dorsoventral polarity in the equal-cleaving spiralian, *Lymnaea palustris* Wilhelm Roux's Arch Dev Biol 1985 194 5 281 295 10.1007/BF01152174

[B42] Henry JJ Conserved mechanism of dorsoventral axis determination in equal-cleaving spiralians Dev Biol 2002 248 2 343 355 10.1006/dbio.2002.0741 12167409

[B43] Dorresteijn AWC Bornewasser H Fischer A A correlative study of experimentally changed first cleavage and Janus development in the trunk of *Platynereis dumerilii* (Annelida, Polychaeta) Roux’s Arch Dev Biol 1987 196 51 58 10.1007/BF00376021 28305659

[B44] Rosenberg MI Lynch JA Desplan C Heads and tails: evolution of antero-posterior patterning in insects Biochim Biophys Acta – Gene Regul Mech 2009 1789 4 333 342 10.1016/j.bbagrm.2008.09.007 PMC2700975 18976722

[B45] Lall S Ludwig MZ Patel NH Nanos plays a conserved role in axial patterning outside of the Diptera Curr Biol 2003 13 3 224 229 10.1016/S0960-9822(03)00045-9 12573218

[B46] Porcher A Dostatni N The bicoid morphogen system Curr Biol 2010 20 5 R249 R254 10.1016/j.cub.2010.01.026 20219179

[B47] Speksnijder JE Terasaki M Hage WJ Jaffe LF Sardet C Polarity and reorganization of the endoplasmic reticulum during fertilization and ooplasmic segregation in the ascidian egg J Cell Biol 1993 120 6 1337 1346 10.1083/jcb.120.6.1337 8449980 PMC2119754

[B48] Nishida H Cell fate specification by localized cytoplasmic determinants and cell interactions in ascidian embryos Int Rev Cytol 1997 176 245 306 9394921 10.1016/s0074-7696(08)61612-5

[B49] Sardet C Paix A Prodon F Dru P Chenevert J From oocyte to 16-cell stage: cytoplasmic and cortical reorganizations that pattern the ascidian embryo Dev Dyn 2007 236 7 1716 1731 10.1002/dvdy.21136 17420986

[B50] Ressom R Dixon K Relocation and reorganization of germ plasm in *Xenopus* embryos after fertilization Development 1988 103 3 507 518 3246221 10.1242/dev.103.3.507

[B51] Whitington PM Dixon K Quantitative studies of germ plasm and germ during early embryogenesis of *Xenopus laevis* J Embryol Exp Morphol 1975 33 1 57 74 1151270

[B52] Strome S Wood WB Generation of asymmetry and segregation of germ-line granules in early C. *elegans* embryos Cell 1983 35 1 15 25 10.1016/0092-8674(83)90203-9 6684994

[B53] Wallenfang MR Seydoux G Polarization of the anterior-posterior axis of *C. elegans* is a microtubule-directed process Nature 2000 408 6808 89 92 10.1038/35040562 11081513

[B54] Freeman G The role of polarity in the development of the hydrozoan planula larva Wilhelm Roux's Arch Dev Biol 1981 190 3 168 184 10.1007/BF00867804 28305168

[B55] Davidson EH Cameron RA Ransick A Specification of cell fate in the sea urchin embryo: summary and some proposed mechanisms Development 1998 125 17 3269 3290 9693132 10.1242/dev.125.17.3269

[B56] Kiyomoto M Shirai H The determinant for archenteron formation in starfish: co-culture of an animal egg fragment-derived cell cluster and a selected blastomere Dev Growth Differ 1993 35 1 99 105 10.1111/j.1440-169X.1993.00099.x 37281945

[B57] Roegiers F McDougall A Sardet C The sperm entry point defines the orientation of the calcium-induced contraction wave that directs the first phase of cytoplasmic reorganization in the ascidian egg Development 1995 121 10 3457 3466 7588078 10.1242/dev.121.10.3457

[B58] Gönczy P Rose LS Asymmetric Cell Division and Axis Formation in the Embryo 2005 WormBook 1 20 10.1895/wormbook.1.30.1 PMC4780927 18050411

[B59] Cowan CR Hyman AA Centrosomes direct cell polarity independently of microtubule assembly in *C. elegans* embryos Nature 2004 431 7004 92 96 10.1038/nature02825 15343338

[B60] Kingsley EP Chan XY Duan Y Lambert JD Widespread RNA segregation in a spiralian embryo Evol Dev 2007 9 6 527 539 10.1111/j.1525-142X.2007.00194.x 17976050

[B61] Lambert JD Nagy LM Asymmetric inheritance of centrosomally localized mRNAs during embryonic cleavages Nature 2002 420 6916 682 686 10.1038/nature01241 12478296

[B62] Lee P Kumburegama S Marlow H Martindale M Wikramanayake A Asymmetric developmental potential along the animal-vegetal axis in the anthozoan cnidarian, *Nematostella vectensis*, is mediated by *Dishevelled* Dev Biol 2007 310 1 169 186 10.1016/j.ydbio.2007.05.040 17716645

[B63] Momose T Derelle R Houliston E A maternally localised Wnt ligand required for axial patterning in the cnidarian *Clytia hemisphaerica* Development 2008 135 12 2105 2113 10.1242/dev.021543 18480163

[B64] Chia W Somers WG Wang H *Drosophila* neuroblast asymmetric divisions: cell cycle regulators, asymmetric protein localization, and tumorigenesis J Cell Biol 2008 180 2 267 272 10.1083/jcb.200708159 18209103 PMC2213578

[B65] Nance J Zallen JA Elaborating polarity: PAR proteins and the cytoskeleton Development 2011 138 5 799 809 10.1242/dev.053538 21303844 PMC3035085

[B66] Ephrussi A Dickinson LK Lehmann R Oskar organizes the germ plasm and directs localization of the posterior determinant nanos Cell 1991 66 1 37 50 10.1016/0092-8674(91)90137-N 2070417

[B67] Kim-Ha J Smith JL Macdonald PM Oskar mRNA is localized to the posterior pole of the *Drosophila* oocyte Cell 1991 66 1 23 35 10.1016/0092-8674(91)90136-M 2070416

[B68] Riechmann V Ephrussi A Axis formation during *Drosophila* oogenesis Curr Opin Genet Dev 2001 11 4 374 383 10.1016/S0959-437X(00)00207-0 11448623

[B69] Chang C-W Nashchekin D Wheatley L Irion U Dahlgaard K Montague TG Hall J Johnston DS Anterior-posterior axis specification in *Drosophila* oocytes: identification of novel bicoid and oskar mRNA localization factors Genetics 2011 188 4 883 896 10.1534/genetics.111.129312 21625003 PMC3176101

[B70] Martin KC Ephrussi A mRNA localization: gene expression in the spatial dimension Cell 2009 136 4 719 730 10.1016/j.cell.2009.01.044 19239891 PMC2819924

[B71] Itoh T Shinagawa A Timing system for the start of gastrulation in the *Xenopus* embryo Dev Growth Differ 2003 45 3 261 273 10.1046/j.1524-4725.2003.692.x 12828687

[B72] Kominami T Takata H Timing of early developmental events in embryos of a tropical sea urchin *Echinometra mathaei* Zoolog Sci 2003 20 5 617 626 10.2108/zsj.20.617 12777832

[B73] Kataoka Y Mishina R Fujiwara S Mechanism of DNA replication-dependent transcriptional activation of the acetylcholinesterase gene in the *Ciona intestinalis* embryo Dev Growth Differ 2009 51 9 841 850 10.1111/j.1440-169X.2009.01147.x 19951326

[B74] Scholtz G Cell lineage studies in the crayfish *Cherax destructor* (Crustacea, Decapoda): germ band formation, segmentation, and early neurogenesis Dev Genes Evol 1992 202 1 36 48 10.1007/BF00364595 28306002

[B75] Satoh N Ikegami S A definite number of aphidicolin-sensitive cell-cyclic events are required for acetylcholinesterase development in the presumptive muscle cells of the ascidian embryos J Embryol Exp Morphol 1981 61 1 1 13 6790652

[B76] Whittaker J Segregation during ascidian embryogenesis of egg cytoplasmic information for tissue-specific enzyme development Proc Natl Acad Sci 1973 70 7 2096 2100 10.1073/pnas.70.7.2096 4198663 PMC433673

[B77] Whittaker J Acetylcholinesterase development in extra cells caused by changing the distribution of myoplasm in ascidian embryos J Embryol Exper Morphol 1980 55 1 343 354 7373203

[B78] Whittaker J Cell lineages and determinants of cell fate in development Am Zool 1987 27 2 607 622

[B79] Zalokar M Effect of colchicine and cytochalasin B on ooplasmic segregation of ascidian eggs Wilhelm Roux Arch Entwickl Mech Org 1974 175 3 243 248 10.1007/BF00582094 28304846

[B80] Deno T Satoh N Studies on the cytoplasmic determinant for muscle cell differentiation in ascidian embryos: an attempt at transplantation of the myoplasm Dev Growth Differ 1984 26 1 43 38 10.1111/j.1440-169X.1984.00043.x 37282166

[B81] Reik W Dean W Walter J Epigenetic reprogramming in mammalian development Science 2001 293 5532 1089 1093 10.1126/science.1063443 11498579

[B82] Newport J Kirschner M A major developmental transition in early *Xenopus* embryos: I. Characterization and timing of cellular changes at the midblastula stage Cell 1982 30 3 675 686 10.1016/0092-8674(82)90272-0 6183003

[B83] Edgar LG McGhee JD DNA synthesis and the control of embryonic gene expression in *C. elegans* Cell 1988 53 4 589 599 10.1016/0092-8674(88)90575-2 3131016

[B84] Tadros W Lipshitz HD The maternal-to-zygotic transition: a play in two acts Development 2009 136 18 3033 3042 10.1242/dev.033183 19700615

[B85] Wang QT Piotrowska K Ciemerych MA Milenkovic L Scott MP Davis RW Zernicka-Goetz M A genome-wide study of gene activity reveals developmental signaling pathways in the preimplantation mouse embryo Dev Cell 2004 6 1 133 144 10.1016/S1534-5807(03)00404-0 14723853

[B86] Wei Z Angerer RC Angerer LM A database of mRNA expression patterns for the sea urchin embryo Dev Biol 2006 300 1 476 484 10.1016/j.ydbio.2006.08.034 17007833 PMC1762123

[B87] Leclère L Jager M Barreau C Chang P Le Guyader H Manuel M Houliston E Maternally localized germ plasm mRNAs and germ cell/stem cell formation in the cnidarian *Clytia* Dev Biol 2012 364 2 236 248 10.1016/j.ydbio.2012.01.018 22309706

[B88] Röttinger E Dahlin P Martindale MQ A Framework for the establishment of a cnidarian gene regulatory network for “endomesoderm” specification: the inputs of β-catenin/TCF signaling PLoS Genet 2012 8 12 e1003164 10.1371/journal.pgen.1003164 23300467 PMC3531958

[B89] Fritzenwanker JH Genikhovich G Kraus Y Technau U Early development and axis specification in the sea anemone *Nematostella vectensis* Dev Biol 2007 310 2 264 279 10.1016/j.ydbio.2007.07.029 17716644

[B90] Dunn CW Hejnol A Matus DQ Pang K Browne WE Smith SA Seaver E Rouse GW Obst M Edgecombe GD Sorensen MV Haddock SHD Schmidt-Rhaesa A Okusu A Mobjerg KR Wheeler WC Martindale MQ Giribet G Broad phylogenomic sampling improves resolution of the animal tree of life Nature 2008 452 745 749 10.1038/nature06614 18322464

[B91] Hejnol A Obst M Stamatakis A Ott M Rouse GW Edgecombe GD Martinez P Baguñà J Bailly X Jondelius U Assessing the root of bilaterian animals with scalable phylogenomic methods Proc Royal Soc B: Biol Sci 2009 276 1677 4261 4270 10.1098/rspb.2009.0896 PMC2817096 19759036

[B92] Wallberg A Thollesson M Farris JS Jondelius U The phylogenetic position of the comb jellies (Ctenophora) and the importance of taxonomic sampling Cladistics 2004 20 6 558 578 10.1111/j.1096-0031.2004.00041.x 34892961

[B93] Leukart R Über die Morphologie und Verwandtschaftsverhältnisse der wirbellosen Tiere 1848 Braunschweig: Vieweg und Sohn

[B94] Nosenko T Schreiber F Adamska M Adamski M Eitel M Hammel J Maldonado M Müller WE Nickel M Schierwater B Vacelet J Wiens M Wörheide G Deep metazoan phylogeny: when different genes tell different stories Mol Phylogenet Evol 2013 67 1 223 233 10.1016/j.ympev.2013.01.010 23353073

[B95] Philippe H Derelle R Lopez P Pick K Borchiellini C Boury-Esnault N Vacelet J Renard E Houliston E Quéinnec E Da Silva C Wincker P Le Guyader H Leys S Jackson DJ Schreiber F Erpenbeck D Morgenstern B Wörheide G Manuel M Phylogenomics revives traditional views on deep animal relationships Curr Biol 2009 19 8 706 712 10.1016/j.cub.2009.02.052 19345102

[B96] Ax P Multicellular Animals: A New Approach to the Phylogenetic Order in Nature, Volume 1 1996 Berlin: Springer

[B97] Layden MJ Meyer NP Pang K Seaver EC Martindale MQ Expression and phylogenetic analysis of the zic gene family in the evolution and development of metazoans EvoDevo 2010 1 1 12 10.1186/2041-9139-1-12 21054859 PMC2988786

[B98] Pang K Ryan JF Baxevanis AD Martindale MQ Evolution of the TGF-β signaling pathway and its potential role in the ctenophore. *Mnemiopsis leidyi* PLoS One 2011 6 9 e24152 10.1371/journal.pone.0024152 21931657 PMC3169577

[B99] Reitzel AM Pang K Ryan JF Mullikin JC Martindale MQ Baxevanis AD Tarrant AM Nuclear receptors from the ctenophore *Mnemiopsis leidyi* lack a zinc-finger DNA-binding domain: lineage-specific loss or ancestral condition in the emergence of the nuclear receptor superfamily EvoDevo 2011 2 1 1 12 10.1186/2041-9139-2-1 21291545 PMC3038971

[B100] Ryan JF Pang K Mullikin JC Martindale MQ Baxevanis AD The homeodomain complement of the ctenophore *Mnemiopsis leidyi* suggests that Ctenophora and Porifera diverged prior to the ParaHoxozoa EvoDevo 2010 1 1 9 10.1186/2041-9139-1-9 20920347 PMC2959044

[B101] Maxwell EK Ryan JF Schnitzler CE Browne WE Baxevanis AD MicroRNAs and essential components of the microRNA processing machinery are not encoded in the genome of the ctenophore *Mnemiopsis leidyi* BMC Genomics 2012 13 1 714 10.1186/1471-2164-13-714 23256903 PMC3563456

[B102] Pick K Philippe H Schreiber F Erpenbeck D Jackson D Wrede P Wiens M Alié A Morgenstern B Manuel M Wörheide G Improved phylogenomic taxon sampling noticeably affects nonbilaterian relationships Mol Biol Evol 2010 27 9 1983 1987 10.1093/molbev/msq089 20378579 PMC2922619

[B103] Dorresteijn A Competence of blastomeres for the expression of molecular tissue markers is acquired by diverse mechanisms in the embryo of *Platynereis* (Annelida) Dev Genes Evol 1993 202 5 270 275 10.1007/BF00363216 28306039

[B104] Nishida H Specification of developmental fates in ascidian embryos: molecular approach to maternal determinants and signaling molecules Int Rev Cytol 2002 217 227 276 12019564 10.1016/s0074-7696(02)17016-1

